# Comment on Zoonoses in the Bedroom

**DOI:** 10.3201/eid1707.110317

**Published:** 2011-07

**Authors:** Susan P. Montgomery, Lihua Xiao, Vitaliano Cama

**Affiliations:** Author affiliation: Centers for Disease Control and Prevention, Atlanta, Georgia, USA

**Keywords:** zoonoses, parasitic diseases, parasites, Chagas disease, *Giardia*, *Cryptosporidium*, *Toxocara*, risk, letter

**To the Editor:** In response to Chomel and Sun (*1*), we would like to correct potentially misleading representations of risk factors for parasitic diseases. The authors correctly described risk for Chagas disease from exposure to infected insect vectors but included Chagas disease in the table, “Zoonoses acquired by close contact with pet, 1974–2010.” The bloodborne protozoan that causes Chagas disease is transmitted not by contact with an infected mammal but by contact with a vector insect that has bitten an infected mammal (*2*).

For some parasitic zoonoses, contact with pets may not be a major source of infection. Molecular studies indicate that risk for human infection with *Giardia* and *Cryptosporidium* spp. from dogs and cats may be lower than previously believed. Infections with these parasites are usually with species-specific genotypes*.* Human infections with assemblages C, D (dog specific), and F (cat specific) of *G. duodenalis* have not been confirmed. Infections with assemblages A or B have been reported for humans and other animal species, including dogs and cats, but no direct transmission has been documented (*3**,**4*). Most human cryptosporidial infections are caused by *C. hominis* and *C. parvum* (*5*); a smaller percentage are caused by *C. canis* and *C. felis.*

Human infection with *Toxocara canis* or *T. cati* occurs when embryonated eggs are ingested; however, embryonation requires 2–4 weeks in the environment, suggesting that the risk from eggs in pet fur may be less than risk from exposure to eggs in contaminated soil. Other more serious zoonotic parasitic disease risks from contact with pet feces, including toxoplasmosis, are mentioned only briefly, if at all.

Physicians need information that accurately communicates zoonotic parasitic disease risks to their patients. However, inaccurate or overstated risk communication can also lead to unnecessary prevention efforts and misdirected concerns about dogs and cats as sources of disease.
