# Supplementary material for: Comment on Zoonoses in the Bedroom
Source: Emerg Infect Dis. 2011 Jul;17(7):1340–1. doi: 10.3201/eid1707.110317 (PMC3381382; doi:10.3201/eid1707.110317)
Supplement: Related texts

# Reply: Comment on Zoonoses in the Bedroom

**Authors:** Bruno B. Chomel, Benjamin Sun

**Affiliations:** Author affiliations: University of California, Davis, California, USA (B.B. Chomel);; California Department of Public Health, Sacramento, California, USA (B. Sun)

**Keywords:** Keywords: zoonoses, pet, bedroom, parasites, letter

**In Response:** We agree with Montgomery et al. (*1*) that Chagas disease is not directly transmitted by dogs to humans. However, we emphasize that Chagas disease is in the southern United States even if risk for infection is extremely low. A study in Mexico found direct correlation of seropositivity between humans and dogs, suggesting that testing dogs may help identify prevalence of *Trypanosoma cruzi* infection among humans. They stated, “Dogs may be domestic reservoir hosts and help maintain human transmission of *T*. *cruzi*” (*2*).

For toxocariasis, indeed only embryonated eggs are infectious. In a study in the Netherlands (*3*), ≈25% of *Toxocara* eggs found on fur were fertilized, but none were viable after 6 weeks; presence of embryonated eggs on dog fur is uncommon but can occur.

We did not mention all zoonoses that could be transmitted in a bedroom, such as toxoplasmosis or ringworm, because we could not identify publications specifically documenting contamination in that environment. We can, however, cite examples of other infections, such as *Cheyletiella blakei* dermatitis in a woman who shared her bed with a recently acquired cat (*4*). We also reiterate the potential risk for human infection by the plague bacillus (*Yersinia pestis*) as a result of bed sharing, as illustrated by the case reported from Oregon in 2010 (*5*).

Although the risk of contracting a zoonosis in the bedroom is low, it remains possible. Bed sharing with pets should be avoided, especially for those who are immunocompromised, young, or elderly.

## References

[1] Montgomery SP, Xiao L, Cama V. Comment on zoonoses in the bedroom [letter]. Emerg Infect Dis. 2011;17:1340. 10.3201/eid1707.11031721762619PMC3381382

[2] Estrada-Franco JG, Bhatia V, Diaz-Albiter H, Ochoa-Garcia L, Barbabosa A, Vazquez-Chagoyan JC, Human *Trypanosoma cruzi* infection and seropositivity in dogs, Mexico. Emerg Infect Dis. 2006;12:624–30.1670481110.3201/eid1204.050450PMC3294681

[3] Overgaauw PA, van Zutphen L, Hoek D, Yaya FO, Roelfsema J, Pinelli E, Zoonotic parasites in fecal samples and fur from dogs and cats in the Netherlands. Vet Parasitol. 2009;163:115–22. 10.1016/j.vetpar.2009.03.04419398275

[4] Tsianakas P, Polack B, Pinquier L, Levy Klotz B, Prost-Squarcioni C. *Cheyletiella* dermatitis: an uncommon cause of vesiculobullous eruption [in French]. Ann Dermatol Venereol. 2000;127:826–9.11060386

[5] Centers for Disease Control and Prevention. Notes from the field: two cases of human plague—Oregon, 2010. MMWR Morb Mortal Wkly Rep. 2011;60:214.21346709
